# Adenosine Stress and Rest T1 Mapping Can Differentiate Between Ischemic, Infarcted, Remote, and Normal Myocardium Without the Need for Gadolinium Contrast Agents

**DOI:** 10.1016/j.jcmg.2015.08.018

**Published:** 2016-01

**Authors:** Alexander Liu, Rohan S. Wijesurendra, Jane M. Francis, Matthew D. Robson, Stefan Neubauer, Stefan K. Piechnik, Vanessa M. Ferreira

**Affiliations:** Division of Cardiovascular Medicine, Radcliffe Department of Medicine, University of Oxford, John Radcliffe Hospital, Oxford, United Kingdom

**Keywords:** adenosine stress, cardiac magnetic resonance, ischemia, ShMOLLI, T1 mapping, CAD, chronic coronary artery disease, CMR, cardiac magnetic resonance, LGE, late gadolinium enhancement, LV, left ventricular, MBF, myocardial blood flow, MBV, myocardial blood volume, ROI, region of interest, ShMOLLI, Shortened Modified Look-Locker Inversion recovery

## Abstract

**Objectives:**

The aim of this study was to evaluate the potential of T1 mapping at rest and during adenosine stress as a novel method for ischemia detection without the use of gadolinium contrast.

**Background:**

In chronic coronary artery disease (CAD), accurate detection of ischemia is important because targeted revascularization improves clinical outcomes. Myocardial blood volume (MBV) may be a more comprehensive marker of ischemia than myocardial blood flow. T1 mapping using cardiac magnetic resonance (CMR) is highly sensitive to changes in myocardial water content, including MBV. We propose that T1 mapping at rest and during adenosine vasodilatory stress can detect MBV changes in normal and diseased myocardium in CAD.

**Methods:**

Twenty normal controls (10 at 1.5-T; 10 at 3.0-T) and 10 CAD patients (1.5-T) underwent conventional CMR to assess for left ventricular function (cine), infarction (late gadolinium enhancement [LGE]) and ischemia (myocardial perfusion reserve index [MPRI] on first-pass perfusion imaging during adenosine stress). These were compared to novel pre-contrast stress/rest T1 mapping using the Shortened Modified Look-Locker Inversion recovery technique, which is heart rate independent. T1 values were derived for normal myocardium in controls and for infarcted, ischemic, and remote myocardium in CAD patients.

**Results:**

Normal myocardium in controls (normal wall motion, MPRI, no LGE) showed normal resting T1 (954 ± 19 ms at 1.5-T; 1,189 ± 34 ms at 3.0-T) and significant positive T1 reactivity during adenosine stress compared to baseline (6.2 ± 0.5% at 1.5-T; 6.3 ± 1.1% at 3.0-T; all p < 0.0001). Infarcted myocardium showed the highest resting T1 of all tissue classes (1,442 ± 84 ms), without significant T1 reactivity (0.2 ± 1.5%). Ischemic myocardium showed elevated resting T1 compared to normal (987 ± 17 ms; p *<* 0.001) without significant T1 reactivity (0.2 ± 0.8%). Remote myocardium, although having comparable resting T1 to normal (955 ± 17 ms; p = 0.92), showed blunted T1 reactivity (3.9 ± 0.6%; p < 0.001).

**Conclusions:**

T1 mapping at rest and during adenosine stress can differentiate between normal, infarcted, ischemic, and remote myocardium with distinctive T1 profiles. Stress/rest T1 mapping holds promise for ischemia detection without the need for gadolinium contrast.

In chronic coronary artery disease (CAD), accurate detection of functional ischemia is important because targeted revascularization improves clinical outcomes 1, 2, 3. First-pass myocardial perfusion cardiac magnetic resonance (CMR) during vasodilatory stress directly assesses reductions in microvascular blood flow (MBF), and has demonstrated high diagnostic accuracy for detecting significant coronary stenosis 1, 2, 3. However, assessment of MBF alone may not reflect all aspects of ischemia 4, 5, 6, 7. Myocardial blood volume (MBV), on the other hand, may be a more comprehensive global marker of ischemia, as it represents the total volume of capacitance vessels in both the microcirculations and macrocirculations 4, 5, 6, 8, 9. Significant coronary artery stenosis induces capillary recruitment with an increase in resting MBV (9). Myocardial blood volume measurements derived from first-pass contrast-based CMR closely reflect the level of microvascular autoregulation 4, 5, 9. As a surrogate for epicardial CAD, recent animal studies showed that disturbances in MBV can effectively detect anatomically significant coronary stenoses 4, 10, and distinguish their functional relevance (11). Moderate and severe coronary stenoses may be better differentiated using the index of myocardial blood volume reserve than by myocardial perfusion imaging (4). Furthermore, MBV may relate better to cardiomyocyte metabolism by reflecting changes in myocardial oxygen consumption, which is a more reliable marker of cellular ischemia 4, 6, 11, 12. Therefore, MBV determination during vasodilatation and at rest may constitute a more complete assessment of ischemia than MBF (via perfusion imaging) alone.

Native (pre-contrast) T1 mapping is a novel CMR technique that can potentially improve ischemia detection by detecting MBV and myocardial water content. In MRI, hydrogen-proton spin-lattice relaxation time (T1) is a magnetic property of tissue that is prolonged by increased water content 13, 14 and, importantly, depends on blood T1 through its partial volume (14). Each tissue type, such as myocardium, has a specific range of normal T1 values, deviation from which is indicative of disease 13, 15, 16, 17, 18, 19, 20. By measuring and displaying T1 relaxation times pixel by pixel, native T1 mapping provides a quantitative biomarker of intracellular and extracellular environments of the myocardium without the need for intravenous contrast agents (13). T1 mapping is highly reproducible with tight normal ranges 13, 14, capable of diagnosing a variety of cardiac diseases 13, 15, 16, 18, 19, 20, 21, 22. Increased myocardial T1 values act as a surrogate for increased myocardial water (13); hence coronary vasodilatation, which increases MBV 4, 5, 6, is expected to prolong T1 and allow detection of microvascular and myocardial blood volume changes during ischemia (9). We have recently demonstrated the ability of stress/rest T1 mapping to detect increases in MBV from coronary vasodilatation in patients with severe aortic stenosis and nonobstructive coronary arteries, with complete reversal and normalization after aortic valve replacement (23). In summary, stress/rest T1 mapping is a highly promising technique for the detection of ischemia and is particularly attractive for applications in patients with CAD.

In this proof-of-principle study, we demonstrate the ability of T1 mapping, during adenosine vasodilatory stress and rest, to distinguish 4 myocardial tissue classes: normal, infarcted, ischemic, and remote myocardium, as a novel gadolinium-free method for ischemia detection. We performed CMR scans in normal controls and patients with known CAD assessing left ventricular (LV) function (cine), viability (late gadolinium enhancement [LGE]), and ischemia (adenosine stress gadolinium first-pass perfusion), and compared them with novel T1 mapping to establish characteristic stress and rest T1 profiles of these 4 myocardial tissues.

## Methods

Ethical approval was granted for all study procedures and all subjects gave written informed consent.

### Normal controls

Twenty normal controls without history of cardiovascular disease, not on cardiovascular medications, and with normal electrocardiograms were recruited. Ten volunteers (7 males, 33 ± 10 years of age) underwent CMR scans at 1.5-T (Avanto, Siemens Healthcare, Erlangen, Germany) and 10 volunteers (7 males, 36 ± 11 years of age) were scanned at 3.0-T (TimTrio, Siemens Medical Solutions), all with identical CMR protocols. All subjects avoided potential adenosine antagonizers (e.g., caffeine) for ≥24 h before CMR scans.

Cine images were obtained as previously described (23). T1 mapping was performed using the Shortened Modified Look-Locker Inversion recovery (ShMOLLI) sequence, which has been shown to be heart rate independent over a wide range of T1 values (13), as previously described 13, 14, 18, 19, 21. In brief, T1 maps were acquired at rest and during peak adenosine stress (140 μg/kg/min, intravenously for ≥3 to 6 min) in 3 short-axis (basal, midventricular, apical) slices before gadolinium administration and first-pass perfusion (23). The basal slice was carefully planned to exclude LV outflow tract. First-pass perfusion imaging was performed, on matching short-axis slices to T1 maps, during peak adenosine stress with an intravenous bolus of gadolinium (0.03 mmol/kg; Dotarem, Guerbet, Villepinte, France), followed by a 15 ml saline flush (12). Rest perfusion images were acquired >15 min after adenosine discontinuation 12, 23. LGE imaging was performed ∼8 to 10 min after an additional bolus of gadolinium (0.1 mmol/kg) (12).

### Patients with CAD

Ten patients with angiographically significant stenosis (>50%) in ≥1 coronary artery, who underwent CMR at 1.5-T using cine, adenosine stress/rest T1 mapping, adenosine stress/rest perfusion, and LGE imaging, were included to illustrate the ability of stress/rest T1 mapping to distinguish myocardial tissue classes.

### Image analysis

LV function and first-pass myocardial perfusion were analyzed as previously described (23). Short-axis T1 maps were manually contoured to outline the endocardium and epicardium using dedicated software and underwent strict and extensive quality control process as previously described 13, 14, 15, 18, 19, 21, 23, which resulted in exclusion of 11.7% of segments (for additional details, see the Online Appendix). For normal controls, mean myocardial T1 values were derived from T1 maps at rest and during adenosine stress per subject, per slice, and per segment (American Heart Association 16-segment model) (21). T1 reactivity to adenosine stress was expressed in absolute terms: ΔT1(ms) = T1_stress_–T1_rest_ and as percentages: δT1(%) = ΔT1÷T1_rest_ × 100. T1_rest_ and T1_stress_ represent mean T1 values at rest and during adenosine stress, respectively.

In CAD patients, the mean T1 of ischemic myocardium was measured by placing a region of interest (ROI) in an area corresponding to the area of reversible perfusion defect on first-pass stress and rest imaging but without LGE, accompanied by angiographic evidence of significant coronary stenosis as assessed by an expert interventional cardiologist. Infarcted myocardium on T1 maps was defined by placing a ROI corresponding to an area of infarction on LGE images, defined as enhancement involving the subendocardium of >50% transmurality, as assessed by 2 experienced observers. To avoid partial volume contamination from the LV blood pool, all infarct ROIs were placed in the core of the infarcts and away from the endocardial and epicardial borders carefully referenced against corresponding cine images in the same phase of the cardiac cycle. Remote myocardial ROI on T1 maps were placed contralateral to the ischemic myocardium in areas without evidence of first-pass perfusion defects, regional wall motion abnormalities, LGE, or significant upstream angiographic coronary stenosis. A reference ROI was also placed in the LV blood pool.

### Statistical analysis

The data are reported as mean ± SD with all tests 2-tailed and parametric, based on Kolmogorov-Smirnov normality checks. Differences in specific individual characteristics, including T1 and relative δT1 in selected types of tissue, are tested using separate Student *t* tests, paired whenever possible in same individuals and unpaired between groups. Repeated measures of interslice and intersegmental δT1 in normal controls were assessed using analysis of variance with Bonferroni corrected post hoc comparisons. Pearson correlation coefficient (R) was used to assess statistical correlation between samples. Unless otherwise stated, all analyses were performed on single measures per-subject. All analyses were performed using MedCalc Software version 12.7.8.0 (Mariakerke, Belgium). All p values <0.05 were considered significant.

## Results

### Myocardial T1 reactivity in normal controls

All 20 normal controls had normal global and regional LV systolic function (LV ejection fraction 66 ± 5%), myocardial perfusion reserve indices (2.0 ± 0.3), and no LGE (Table 1).

**Table 1:** Characteristics of Study Subjects

	1.5-T		3.0-T
	Patients (n = 10)	Controls (n = 10)	Controls (n = 10)
Male	8 (80)	7 (70)	7 (70)
Age, yrs	64 ± 11∗	32 ± 10	36 ± 11
Body mass index, kg/m^2^	28 ± 4∗	22 ± 2	25 ± 3
Resting heart rate, beats/min	61 ± 8	68 ± 12	64 ± 15
Stress heart rate, beats/min	85 ± 9∗	103 ± 11	95 ± 20
Systolic blood pressure, mm Hg	125 ± 9	114 ± 13	125 ± 20
Diastolic blood pressure, mm Hg	76 ± 11	73 ± 10	80 ± 11
Rest rate pressure product, mm Hg·beats/min	7,700 ± 1,400	7,800 ± 1,400	8,100 ± 2,900
Stress rate pressure product, mm Hg·beats/min	10,700 ± 1,700	11,600 ± 1,800	11,900 ± 4,000
Hematocrit, %	46 ± 7	—	—
CMR data			
Left ventricular ejection fraction, %	55 ± 16	66 ± 5	66 ± 5
Myocardial perfusion reserve index	1.9 ± 0.5 (remote) 1.1 ± 0.3 (ischemic)∗	2.0 ± 0.2	2.0 ± 0.4
Late gadolinium enhancement, %	16 ± 9∗	—	—

Values are n (%) or mean ± SD.

CMR = cardiac magnetic resonance.

∗ p *<* 0.05 compared to 1.5-T controls.

**Figure 1:**
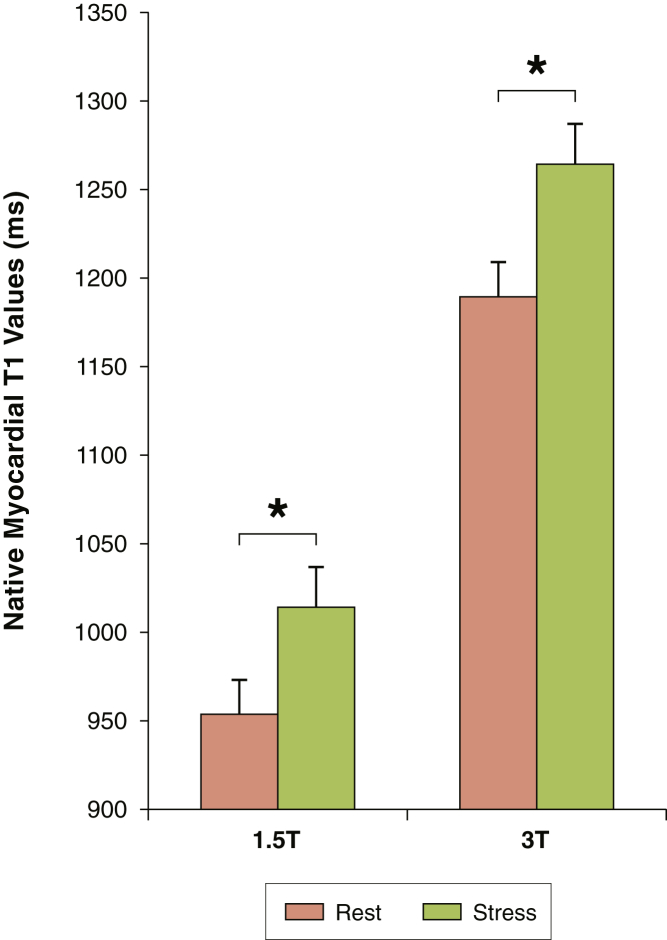
Comparison of Native Myocardial T1 Values at Rest and During Adenosine Stress in Normal Controls at 1.5- and 3.0-T Resting myocardial T1 values were normal: 1.5-T (954 ± 19 ms) and 3.0-T (1,189 ± 34 ms). During adenosine stress, T1 values increased significantly at 1.5-T (59 ± 5 ms/6.2 ± 0.5%) and 3.0-T (75 ± 14 ms/6.3 ± 1.1%; all p < 0.0001), with no significant differences in T1 reactivity between the 2 field-strengths (p = 0.60). All bars represent mean T1 values (ms) ± 1 SD. *p < 0.0001.

**Figure 2:**
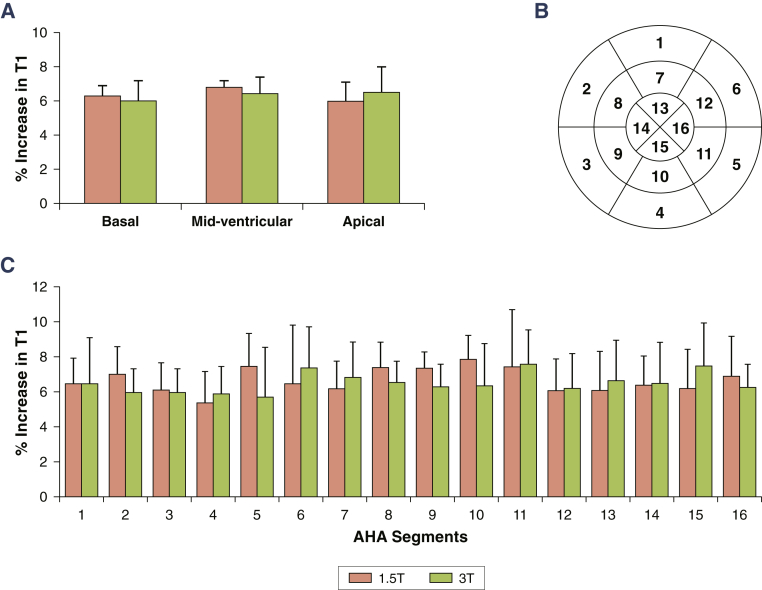
Per-Slice and Per-Segmental Percentage Myocardial T1 Reactivity to Adenosine Stress (δT1) in Normal Controls at 1.5- and 3.0-T **(A)** δT1 showed no significant variations between basal, midventricular, and apical slices at 1.5-T (6.3 ± 0.6% vs. 6.7 ± 0.4% vs. 6.0 ± 1.0%; p = 0.13) or 3.0-T (6.0 ± 1.2% vs. 6.4 ± 1.0% vs. 6.5 ± 1.5%; p = 0.64). Analysis according to the American Heart Association 16-segment model **(B)** showed that δT1 was independent of segment positioning at 1.5-T and 3.0-T (p = 0.95 and p = 0.93, respectively) **(C)**. All bars represent mean δT1(%) ± 1 SD.

Normal control T1 values at rest were within previously published ranges for ShMOLLI: 954 ± 19 ms (1.5-T) and 1,189 ± 34 ms (3.0-T) 13, 14, 17. Compared to rest, myocardial T1 values during adenosine stress increased significantly at 1.5-T (+59 ± 5 ms) and 3.0-T (+75 ± 14 ms; both p *<* 0.0001) (Figure 1). There were no significant differences in δT1 between the 2 field strengths (δT1 = 6.2 ± 0.5% vs. 6.3 ± 1.1%, respectively; p *=* 0.60). Similarly, there were no significant differences between interslice and intersegment δT1 values (p > 0.13, ANOVA) despite apparent per-segment variations in resting myocardial T1 values as previously described (14) (Figure 2B). There was no significant correlation between age and T1 reactivity in the normal controls at 1.5-T (R^2^ = 0.077, p *=* 0.43) or 3.0-T (R^2^ = 0.035, p *=* 0.60), over a wide age range (21 to 57 years of age).

### Myocardial T1 reactivity in CAD patients

Ten patients with known CAD underwent CMR at 1.5-T (LV function, adenosine stress/rest T1 mapping, and first-pass perfusion, LGE). Each patient demonstrated ≥1 reversible perfusion defect indicative of inducible ischemia on first-pass perfusion imaging, referenced to ≥1 angiographically significant coronary lesion (>50%) and without LGE. Additionally, all 10 patients showed evidence of previous myocardial infarction on LGE remote from the ischemic territories (Table 2).

**Table 2:** Characteristics of Patients (n = 10) With Coronary Artery Disease

Male	8 (80)
Age, yrs	64 ± 11
Risk factors	
Current smoker	1 (10)
Ex-smoker	5 (50)
Hypertension	6 (60)
Hypercholesterolemia	5 (50)
Diabetes mellitus	3 (30)
Family history of ischemic heart disease	4 (40)
Medications	
Aspirin	10 (100)
Beta-blocker	10 (100)
ACE inhibitor/angiotensin receptor blocker	5 (50)
Statin	6 (60)
Calcium-channel blocker	3 (30)
Nitrates	3 (30)
CMR data	
≥1 perfusion defect(s) referenced to angiographic stenosis	10 (100)
Full thickness late gadolinium enhancement	4 (40)
Partial thickness late gadolinium enhancement	6 (60)
Age of chronic myocardial infarctions, months	71 ± 70
Angiographic data	
≥1 lesion (>50% visual diameter stenosis)	10 (100)
Left anterior descending artery	5 (40)
Left circumflex artery	4 (50)
Right coronary artery	6 (60)

Values are n (%) or mean ± SD.

ACE = angiotensin-converting enzyme; CMR = cardiac magnetic resonance.

**Figure 3:**
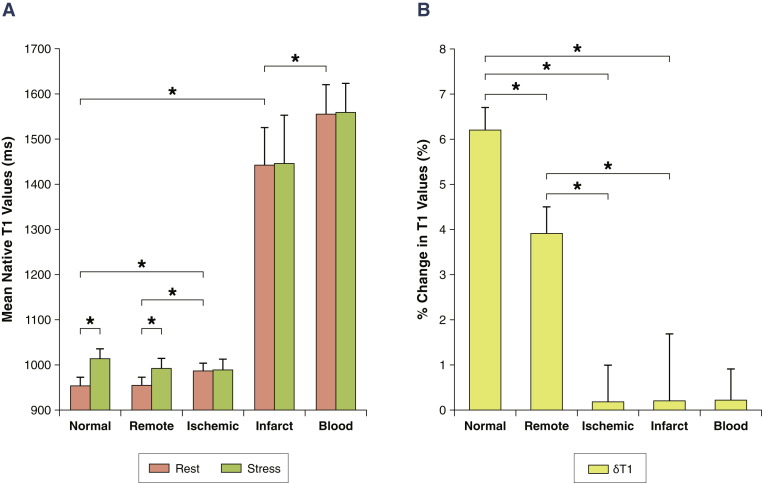
Myocardial T1 at Rest and During Adenosine Stress at 1.5-T **(A)** T1 values at rest in normal and remote tissue were similar and significantly lower than in ischemic regions. Infarct T1 was the highest of all myocardial tissue, but lower than the reference left ventricular blood pool of patients. During adenosine stress, normal and remote myocardial T1 increased significantly from baseline, while T1 in ischemic and infarcted regions remained relatively unchanged. **(B)** Relative T1 reactivity (δT1) in the patient’s remote myocardium was significantly blunted compared to normal, and completely abolished in ischemic and infarcted regions. All data indicate mean ± 1 SD. *p < 0.05.

At rest, the remote myocardial T1 (955 ± 17 ms) was comparable to normal controls at 1.5-T (954 ± 19 ms; p *=* 0.92). Ischemic myocardial T1 (987 ± 17 ms) was significantly higher than normal and remote myocardial T1 (all p < 0.001). T1 in infarcted myocardium (1,442 ± 84 ms) was substantially longer than in any other myocardial tissue (all p < 0.001) (Figure 3A), and did not correlate with remote (R = –0.34, p = 0.34) or ischemic (R = 0.10, p = 0.79) myocardial T1. Moreover, T1 in infarcted myocardium was significantly shorter than in LV blood pool (1,555 ± 66 ms; p = 0.003), with no significant correlations between the 2 (R = 0.14, p = 0.69), supporting the notion that potential contamination of the infarct T1 by partial volume effects from the LV blood pool is minimal.

There was no significant stress δT1 reactivity in ischemic (0.2 ± 0.8%) or infarcted (0.2 ± 1.5%) myocardium compared to controls at 1.5-T (all p < 0.001). Although remote myocardial δT1 reactivity (3.9 ± 0.6%) was greater than in ischemic and infarcted myocardium (all p < 0.001), it was significantly blunted compared to normal controls at 1.5-T (6.2 ± 0.5%; all p < 0.001) (Figure 3B). No significant δT1 reactivity was observed in the LV blood pool (0.21 ± 0.7%).

To check for potential confounding effects of vasodilator medications on myocardial T1, the baseline T1 of patients on nitrate therapy (n = 7) was compared to patients without (n = 3), and no significant baseline T1 differences were observed in the remote (953 ± 16 ms vs. 959 ± 6 ms, respectively; p = 0.39), ischemic (986 ± 16 ms vs. 989 ± 9 ms, respectively; p = 0.26), or infarcted (1,436 ± 114 ms vs. 1,455 ± 83 ms, respectively; p = 0.39) myocardium between the 2 patient groups. Myocardial tissue characteristics are summarized in Tables 3 and 4.

**Table 3:** CMR Characteristics of Normal, Remote, Ischemic, and Infarcted Myocardial Tissue

	Normal Controls (1.5-T)	Patients With CAD (1.5-T)		
		Remote	Ischemic	Infarct
Regional wall motion abnormalities	–	–	+	+
Myocardial perfusion reserve index	2.0 ± 0.2	1.9 ± 0.5	1.1 ± 0.3	1.0 ± 0.2
Late gadolinium enhancement	–	–	–	+
T1 value at rest, ms	954 ± 19	955 ± 17	987 ± 17	1,442 ± 84
T1 value at peak adenosine stress, ms	1,013 ± 23	992 ± 22	989 ± 24	1,445 ± 107
δT1 (% stress reactivity compared to rest)	6.2 ± 0.5	3.9 ± 0.6	0.2 ± 0.8	0.2 ± 1.5

Values are mean ± SD.

CAD = chronic coronary artery disease; CMR = cardiac magnetic resonance.

**Table 4:** T1 Profiles of Myocardial Tissue Based on Rest and Stress Native T1 Mapping

	Normal Controls	Patients With CAD		
		Remote	Ischemic	Infarct
Resting T1	Normal	Normal		
δT1 (% stress reactivity compared to rest)	(∼6%)	(∼4%)	(<1%)	(<1%)

CAD = chronic coronary artery disease.

**Figure 4:**
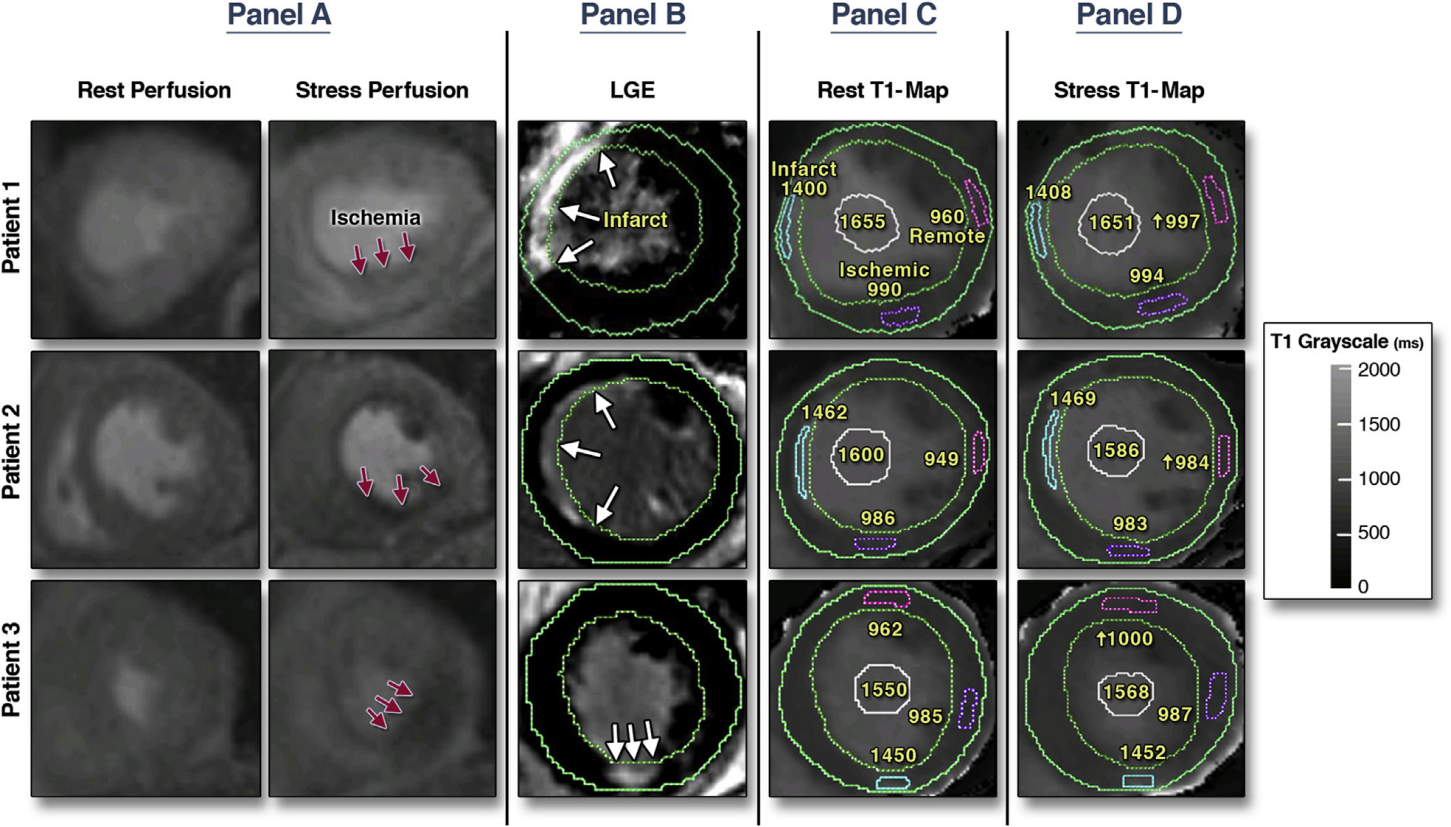
CMR Assessment of 3 Patients With Coronary Artery Disease Using Adenosine Stress/Rest T1 Mapping, Stress/Rest Gadolinium Perfusion, and Late Gadolinium Enhancement Imaging Each patient showed an area of inducible ischemia on stress/rest perfusion images **(A, red arrows)** and an area of infarction **(B, white arrows)**. On T1 maps **(C and D)**, the corresponding remote, ischemic, and infarcted regions as well as the LV blood pool are as labeled. In all 3 patients, the remote myocardial T1 at rest was within normal ranges (14), which increased significantly with adenosine stress (marked by ↑). No significant T1 reactivity was observed in the ischemic or infarcted regions, or the left ventricular blood pool. Reference color T1 maps are shown in the Online Figure 1 for comparative/illustrative purposes. CMR = cardiac magnetic resonance.

Figure 4 shows examples of 3 CAD patients with inducible ischemia on first-pass perfusion imaging, myocardial infarctions on LGE and localized corresponding changes on stress/rest T1 maps. Color T1-maps are shown in Online Figure 1.

## Discussion

This proof-of-principle study demonstrates for the first time that normal, remote, and ischemic myocardium have distinctive ranges of T1 reactivity to adenosine vasodilatory stress that can be detected by T1 mapping without gadolinium contrast administration. The finding that T1 reactivity is blunted in remote myocardium of CAD patients compared to normal controls may provide novel insights into disease characteristics of the remote myocardium. Significantly higher resting T1 in ischemic myocardium compared to normal controls and remote regions may potentiate detection of ischemia without vasodilatory stress, in the absence of other causes of T1 elevations at rest 16, 17, 18, 20, 22, 24.

### Myocardial blood volume as a biomarker for ischemia

Myocardial ischemia occurs when oxygen supply is inadequate to meet tissue metabolic needs, which is governed by MBF and MBV 7, 10. Myocardial oxygen extraction is near maximal at rest and any increase in oxygen demand is met by an increase in MBF via coronary arteriole vasodilatation and capillary recruitment (7). While the relationships between vascular diameter, flow and blood volume are not straightforward due to differences in reactivity between vessel types (8), MBV and MBF are clearly related and both offer insights into myocardial vascular reserve. Indeed, contrast echocardiography studies demonstrated nonlinear relations between MBV and MBF, especially at higher levels of tissue oxygen consumption 4, 5, 6. Recent studies also showed that MBV is reactive to both vasodilatory and inotropic pharmacological agents in coronary artery disease 4, 5, 6, with the ability to delineate the functional relevance of coronary stenosis (11).

### Potential uses of adenosine stress and rest T1 mapping as a diagnostic tool for ischemia

From the perspective of CMR as a diagnostic imaging tool for ischemia, an increase in MBV is expected to increase the myocardial water content and prolong T1 relaxation time. T1 mapping offers highly reproducible pixel-wise estimations of myocardial T1, both in normal and pathologic states 15, 16, 17, 18, 19, 20, 21, 24. Approximately 10% of the myocardial voxel content is occupied by blood volume, which therefore contributes to the myocardial T1 (14) and we now confirm the clear observation of myocardial T1 responses to adenosine vasodilatory stress.

In addition to the stress myocardial T1 response, resting myocardial T1 may also offer additional insights into coronary vascular reserve and function. Notably, ischemic myocardium in CAD patients demonstrate resting T1 values that are nearly as high as in controls during vasodilatory stress, and showed no further reactivity to stress. This strongly suggests that observed elevation in resting T1 in remote myocardium may originate from the intravascular compartment. This may be consistent with maximal compensatory coronary microvascular vasodilatation or increased capillary recruitment downstream from angiographically significant stenosis to maintain adequate tissue oxygen extraction in the face of chronic ischemia (3).

Another interesting point of note is that, in CAD patients, while remote and ischemic myocardium demonstrated the same maximal T1 during stress, this is blunted when compared to controls during maximal vasodilation. Arnold et al. (12) recently demonstrated blunted absolute myocardial perfusion and stress/rest blood oxygen level dependence responses in remote myocardium in a similar patient group to our study, thought to be due to coronary microvascular dysfunction. The origins of the increased resting T1 and the blunted maximal stress T1 response in remote myocardium of patients with CAD compared to normal controls may reflect a degree of microvascular dysfunction, although this is speculative and deserves further investigation.

In this first proof-of-principle study, we relied on widely accepted gadolinium-based CMR techniques (first-pass perfusion and LGE imaging) to define 4 myocardial tissue classes and to demonstrate that normal, infarcted, ischemic, and remote myocardium have distinct stress/rest T1 value profiles that allow their differentiation from each other. Currently, the use of T1 maps alone for immediate visual diagnostics is challenged by intersegmental T1 variations, relatively small “blood versus infarct” T1 differences and interindividual variations in blood T1 13, 14; with further work and clinical validation, however, this proof-of-principle study paves the way for the eventual development of an automated system using stress/rest T1 values that enables clinicians to visually detect ischemic, remote, and infarcted myocardium without the need for gadolinium contrast imaging.

### Native T1 of chronic myocardial infarctions

The chronic infarct T1 observed in this study is higher than reported in the literature (25). Because this can only partially be attributed to the known heart rate–dependent underestimation of long T1 values by Modified Look-Locker Inversion Recovery (MOLLI) techniques 13, 26, a couple of other explanations are possible. Because the infarcts are subendocardial, there are likely some partial volume effects from the LV blood pool contributing to the high infarct T1. In this study, to estimate the reference T1 for true chronic infarcts, each infarct ROI was placed at the core of the infarct lesion, minimizing partial volume effects of LV blood pool and the surrounding myocardium as much as possible. This is in contrast to previous studies, which either reported T1 values of the entire planimetric infarct area (25) or of the whole myocardial segment (containing both infarct and surrounding myocardium) (15). While infarct T1 may be contaminated by higher LV blood pool T1 or lower surrounding myocardial T1, there were no significant correlations between these tissues in this study. Moreover, an interesting observation is that the mean infarct age in our patients (71 ± 70 months) was higher than typically reported in the literature (usually ∼6 months) 13, 15, 25. Although myocardial scars form within 2 to 3 months post-infarction, the fibrotic process is known to continue beyond 6 months and may take years to complete (27). It is possible that the higher observed infarct T1 in this study, in the absence of lipomatous metaplasia, may potentially represent more extensive fibrotic remodeling with perhaps greater interstitial space expansion for potential water accumulation (leading to higher T1). However, this hypothesis cannot be fully addressed by the data in this study alone. The validation of chronic infarct T1 would strongly benefit from further studies to assess the impact of the partial volume effect, segmental versus regional ROI analysis, and infarct chronicity.

### Adenosine stress/rest T1 mapping and heart rate variations

Adenosine stress can significantly increase the human heart rate by up to 30 to 40 beats/min in normal controls 1, 2, 3, 12, 23, which is a known confounder for many MOLLI-based T1 mapping techniques 13, 25, 26. We previously demonstrated that T1 estimation using the ShMOLLI technique is independent of heart rate variations (40 to 100 beats/min) over the applicable range of T1 values in phantoms and simulations at 1.5- and 3.0-T (13). Hence, any potential confounding effects due to technical heart rate dependencies on the findings in this study are negligible, and the observed T1 values most likely reflect true physiologic and pathophysiologic changes in controls and patients, respectively. Given the multiple variants of available T1 mapping techniques, it is paramount that studies investigating relationships between resting heart rates and T1 between individuals (14) or stress heart rate responses within individuals are performed using T1 mapping techniques that are proven to be technically stable over the required T1 and heart rate ranges (28). Overall, the relationship between stress/rest T1 estimation and heart rate is an interesting topic that requires further validation comparing different T1 mapping techniques.

### Study limitations

This small proof-of-principle study serves to illustrate the potential of stress and rest T1 mapping in differentiating myocardial tissue classes, and will benefit from a larger cohort to systematically investigate potential age or gender differences in myocardial T1 reactivity to vasodilatory stress. Our normal controls were younger and with a male predominance; however, comparing our findings to those of Mahmod et al. (23) (n = 16 normal controls, 50% male, 63.3 ± 3.4 years of age, rest T1 1,168 ± 27 ms, stress T1 1,238 ± 54 ms, T1 reactivity 6.0 ± 4.2%, 3-T), the normal T1 reactivity of ∼6% appears to be independent of age and field strength. The tight normal range of T1 reactivity we observed also supports that any potential bias in the method would be small. Although hematocrit values were within normal ranges in CAD patients in this study, larger hematocrit variations may be observed in wider clinical populations, which may confound native T1 measures and should be further investigated. Larger clinical and population-based studies would be useful to produce standardized ranges for δT1 and to further investigate any effects of unknown confounders. In this study, ROI were drawn away from the subendocardium to minimize partial volume effects; however, placement of ROI closer to the blood pool may be better for detection of subendocardial ischemia. In addition, patients with known and/or high pre-test probability for significant CAD were selected to show typical differences in T1 reactivity between ischemic, remote and infarcted myocardial tissues; future studies with blinded analysis are needed to fully establish sensitivities, specificities, and overall diagnostic accuracy of T1 mapping in patients with a range of pre-test probabilities for CAD, smaller subendocardial infarctions, mixed tissue classes (e.g., peri-infarct ischemia), and including correlations with invasive measures, such as coronary or fractional flow reserves.

## Conclusions

Adenosine stress and rest T1 mapping can differentiate between normal, infarcted, ischemic, and remote myocardial tissue classes with distinctive T1 profiles. Stress/rest T1 mapping on CMR holds promise for ischemia detection without the need for gadolinium contrast.

Perspectives **COMPETENCY IN MEDICAL KNOWLEDGE:** Adenosine stress and rest T1 mapping is a novel CMR technique that reflects changes in myocardial blood volume through its water content. This proof-of-principle study showed for the first time that stress and rest T1 mapping can differentiate between normal, infarcted, ischemic, and remote myocardium with distinctive T1 profiles, which holds promise for ischemia detection using CMR without the need for gadolinium contrast agents.

**TRANSLATIONAL OUTLOOK:** Future studies are needed to establish the diagnostic performance of stress/rest T1 mapping for detecting ischemia in patients with a range of pre-test probabilities for CAD compared to other tests for ischemia detection, and whether information provided by stress/rest T1 mapping can successfully guide targeted revascularization to improve clinical outcomes and long-term survival of patients with CAD.
