# Supplementary material for: Adenosine Stress and Rest T1 Mapping Can Differentiate Between Ischemic, Infarcted, Remote, and Normal Myocardium Without the Need for Gadolinium Contrast Agents
Source: JACC Cardiovasc Imaging. 2016 Jan;9(1):27–36. doi: 10.1016/j.jcmg.2015.08.018 (PMC4708879; doi:10.1016/j.jcmg.2015.08.018)
Supplement: Online Data [file mmc1.docx]

**Online Appendix**

**Methods**

*Image analysis - T1-map image post-procession and quality control*

Short-axis T1-maps were manually contoured using in-house software MC-ROI (programmed by S.K.P. in Interactive Data Language, version 6.1, Exelis Visual Information Solutions, Boulder, Colorado) ([1-6](#_ENREF_1)) to outline the endocardium and epicardium, carefully avoiding contamination by blood-pool and extra-myocardial structures to minimise partial volume effect. Accuracy of the contours were cross-referenced against cine imaging, and for T1-maps, contours were also checked against all available original IR-weighted images. T1-maps were assessed for quality as previously published by examination of the T1-maps, raw T1 images and R^2^ maps ([1-3](#_ENREF_1)).

**Results**

*Myocardial T1-reactivity in normal controls - T1-map inclusion and exclusion for data analysis*

In total, 360 short-axis slices of T1-maps were acquired (180 at rest; 180 under adenosine stress) and subsequently divided into 1920 segments according to the AHA 16-segment model. Strict criteria were applied in the inclusion of only excellent quality segments for the purposes of establishing normative values in this study, excluding 11.7% of the segments due to off-resonance artifacts, partial volume effect, poor T1 fit on the R^2^ maps, patient movement or low signal-to-noise, consistent with previously published data using the same T1-mapping method ([1](#_ENREF_1)). 1696 segments were included in the final analysis.

**Appendix Figure 1: Figure 4 with Reference color T1-maps for comparative/illustrative purposes**


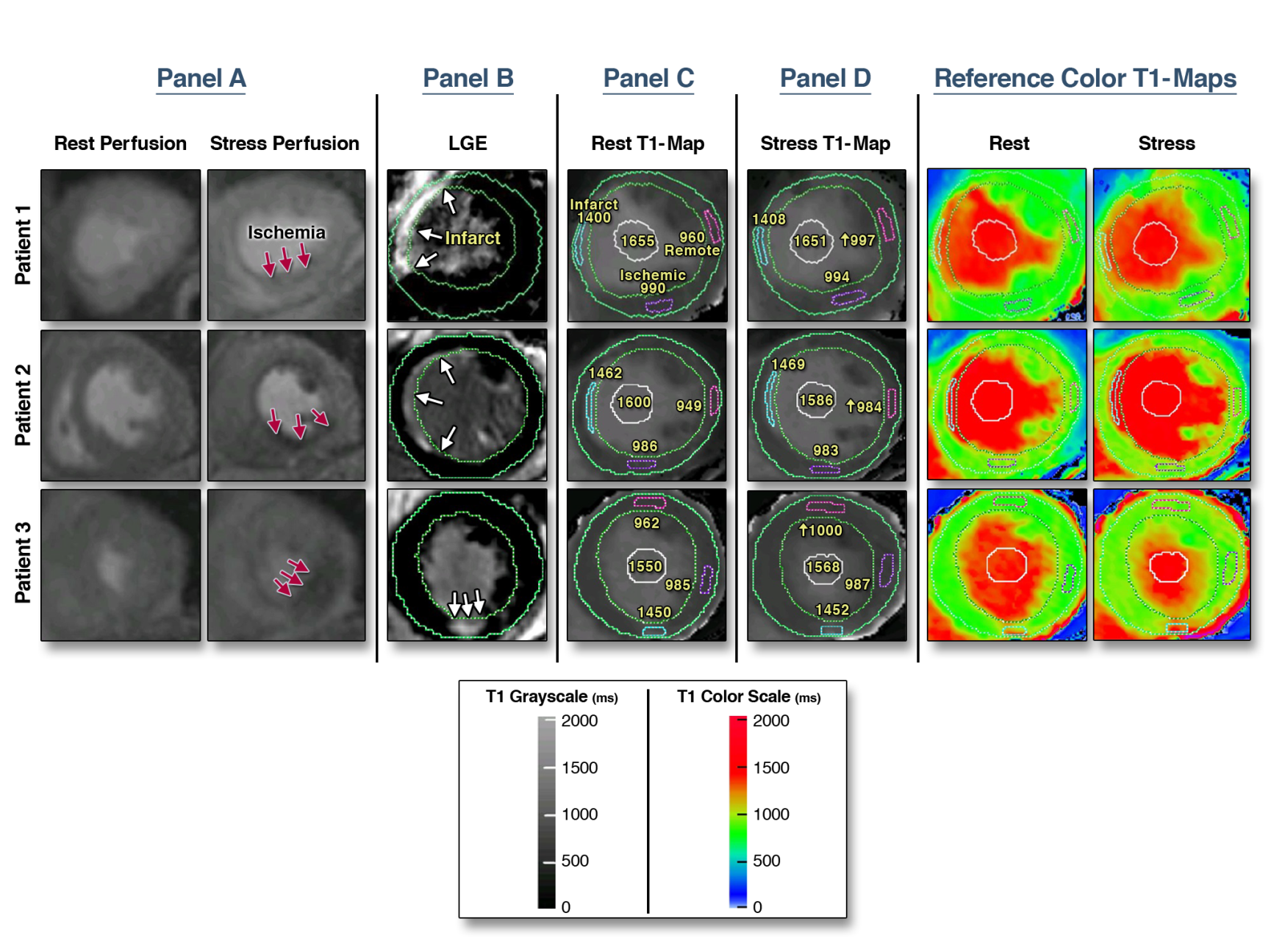


**CMR assessment of 3 patients with coronary artery disease using adenosine stress/rest T1-mapping, stress/rest gadolinium perfusion and late gadolinium enhancement (LGE) imaging.** Each patient showed an area of inducible ischemia on stress/rest perfusion images (Panel A, red arrows) and an area of infarction (Panel B, white arrows). On T1-maps, (Panels C and D), the corresponding remote, ischemic, infarcted regions and the LV blood pool are as labelled. In all 3 patients, the remote myocardial T1 at rest was within normal ranges ([14](#_ENREF_14)), which increased significantly with adenosine stress (marked by ↑). No significant T1-reactivity was observed in the ischemic, infarcted regions or the LV blood pool. Reference color T1-maps are shown for comparative/illustrative purposes. The color T1-maps are displayed using the standard color scheme for ShMOLLI distributed on Siemens CMR scanners (WIP 561, 488C and 780B).

**References**

1. Ferreira VM, Piechnik SK, Dall'Armellina E et al. Non-contrast T1-mapping detects acute myocardial edema with high diagnostic accuracy: a comparison to T2-weighted cardiovascular magnetic resonance. J Cardiovasc Magnetic Resonance 2012;14:42.

2. Ferreira VM, Piechnik SK, Dall'armellina E et al. T1 Mapping for the Diagnosis of Acute Myocarditis Using CMR: Comparison to T2-Weighted and Late Gadolinium Enhanced Imaging. JACC Cardiovascular imaging 2013;6:1048-58.

3. Puntmann VO, D'Cruz D, Smith Z et al. Native myocardial T1 mapping by cardiovascular magnetic resonance imaging in subclinical cardiomyopathy in patients with systemic lupus erythematosus. Circ Cardiovasc Imaging 2013;6:295-301.

4. Dall'Armellina E, Piechnik SK, Ferreira VM et al. Cardiovascular magnetic resonance by non contrast T1-mapping allows assessment of severity of injury in acute myocardial infarction. J Cardiovasc Magnetic Resonance 2012;14:15.

5. Piechnik SK, Ferreira VM, Lewandowski AJ et al. Normal variation of magnetic resonance T1 relaxation times in the human population at 1.5 T using ShMOLLI. J Cardiovasc Magnetic Resonance 2013;15:13.

6. Mahmod M, Piechnik SK, Levelt E et al. Adenosine stress native T1 mapping in severe aortic stenosis: evidence for a role of the intravascular compartment on myocardial T1 values. J Cardiovasc Magnetic Resonance 2014;16:92.
